# Incidence Trends of Urinary Bladder and Kidney Cancers in Urban Shanghai, 1973-2005

**DOI:** 10.1371/journal.pone.0082430

**Published:** 2013-12-04

**Authors:** Yang Yang, Li Xie, Jia-Li Zheng, Yu-Ting Tan, Wei Zhang, Yong-Bing Xiang

**Affiliations:** 1 Department of Epidemiology, Shanghai Cancer Institute, Renji Hospital, Shanghai JiaoTong University School of Medicine, Shanghai, China; 2 State Key Laboratory of Oncogene and Related Genes, Shanghai Cancer Institute, Renji Hospital, Shanghai JiaoTong University School of Medicine, Shanghai, China; 3 Biostatistics Department, College of Public Health, University of Nebraska Medical Center, Omaha, Nebraska, United States of America; University of Campinas, Brazil

## Abstract

**Objectives:**

We examined the incidence trends of bladder and kidney cancers using a population-based cancer registration data.

**Methods:**

Age-standardized incidence rates were analyzed using data from the Shanghai Cancer Registry during 1973 to 2005. Annual percentage changes and 95% confidence intervals were calculated to evaluate the incidence changes. Age-period-cohort analysis was further implemented to assess the contributions of age, period and cohort effects to the trends using the intrinsic estimator method.

**Results:**

In total, 12,676 bladder and 5,811 kidney cancer patients were registered in urban Shanghai. The age-standardized rates of bladder cancer in males increased from 6.39 to 7.66 per 100,000, or 0.62% per year, whereas the rates in females increased from 1.95 to 2.09 per 100,000, or 0.33% per year. For kidney cancer, the age-standardized rates in males increased from 1.20 to 5.64 per 100,000, or 6.98% per year. Similarly in females, the rates increased from 0.85 to 3.33 per 100,000, or 5.93% per year. Age-period-cohort analysis showed increasing curves of age and period effects but generally decreasing cohort effects for bladder and kidney cancers.

**Conclusions:**

Our results show increasing incidence trends of bladder and kidney cancers in Chinese men and women, especially for kidney cancer.

## Introduction

Nowadays, urinary bladder and kidney cancers are the most frequent malignant tumors of the urinary tract, together making up about 5% of all the cancers worldwide. And it also represents the 11^th^ and 14^th^ most common cancers respectively in terms of estimated age-standardized incidence rates [1]. In China, there were an estimated 54,927 new cases and 21,024 deaths of bladder cancer, and 32,508 new cases and 10,675 deaths of cancers of kidney, renal pelvis and ureter in 2008, respectively [1].

Earlier studies conducted in Western countries indicated that both urinary bladder and kidney cancers showed the increasing incidence trends in past several decades [2-5]. However, there has been little information about incidence trends of these two cancers in the Asian population so far. Thus, in order to have comprehensive knowledge of incidence trends of urinary bladder and kidney cancers in China, we conducted a time trend analysis on these cancers using data from the population-based Shanghai Cancer Registry.

## Materials and Methods

### Cancer patient and general population data

The Shanghai Cancer Registry, an associate member of the International Agency for Research on Cancer (IARC), is one of the largest population-based Cancer Registries in developing world. The data which is collected, processed and reported systematically by using standard procedures has been published in consecutive volumes of the Cancer Incidence of Five Continents series by IARC. The study populations were residents of all the ten original urban districts of Shanghai, namely Huang Pu, Nan Shi, Lu Wan, Xu Hui, Chang Ning, Jing An, Pu Tuo, Zha Bei, Hong Kou and Yang Pu districts. Male and female populations in urban area were obtained from the Shanghai Municipal Bureau of Public Security at the end of each year during 1973 to 2005. The mid-year population by sex was estimated based upon the populations at the ends of two consecutive years provided by Shanghai Municipal Bureau of Public Security and used as estimate of the annual average population. Based on eight national or local city censuses, information on population of urban area by sex-age group for census years was obtained. The population in the sex-age group for the remaining years between two consecutive censuses were estimated via linear interpolation method [6].

According to the regulation issued by the Shanghai Municipal Bureau of Public Health, all medical facilities in Shanghai are responsible for notifying all newly diagnosed cancer cases to the Shanghai Cancer Registry. In this study, all newly-diagnosed incidence cases of cancers of bladder, kidney, renal pelvis and ureter from 1973 to 2005 were registered in the Shanghai Cancer Registry. The calendar periods of 1973–2001 and 2002–2005 were respectively collected by the Shanghai Cancer Institute and the Shanghai Center for Disease Control and Prevention. The codes for different urinary tract cancer sites were converted into 10^th^ revision of the International Classification of Diseases (ICD-10), i.e. C64 for malignant neoplasm of kidney, except renal pelvis (hence forth referred to as “kidney cancer” for short), C65 for malignant neoplasm of renal pelvis, C66 for malignant neoplasm of ureter, C67 for malignant neoplasm of bladder and C68 for malignant neoplasm of other and unspecified urinary organs. More detail information on the Shanghai Cancer Registry could be obtained from our previously published papers and monograph [6-8].

The data quality was depend on the diagnostic criteria of histological verification (HV) which included histological detection and cytological or biochemical detection [9]. The proportions of patients diagnosed by surgery or medical imaging such as B ultrasound, X-ray, computed tomography were also calculated in our analysis. Moreover, the proportions of clinical deduction and death certificate (It included the death certificate notification (DCN) and death certificate only (DCO) cases before 1987; And it was only the DCO cases after 1987.) were also used to present the quality of our data (Table 1).

**Table 1:** Proportions (%) of diagnostic evidence for urinary bladder and kidney cancers in urban Shanghai (1973-2005).

**Sites & Periods**	**No. of cases**	**Histological detection**	**Cytological or biochemical detection**	**Surgery or medical imaging**	**Clinical deduction**	**Death certificate^*a*^**	**Unknown**
Bladder cancer							
1973-1975	651	45.78	0.00	17.51	4.92	31.80	0.00
1976-1978	732	54.37	0.41	13.25	2.05	29.92	0.00
1979-1981	786	61.20	0.64	11.20	3.82	23.16	0.00
1982-1984	875	61.94	1.49	8.80	4.46	23.31	0.00
1985-1987	1055	64.64	1.90	9.67	3.89	19.81	0.09
1988-1990	1105	72.58	1.09	17.56	4.34	4.34	0.09
1991-1993	1260	72.38	1.43	21.90	4.29	0.00	0.00
1994-1996	1258	74.96	0.64	21.14	3.18	0.00	0.08
1997-1999	1487	76.66	0.47	20.85	2.02	0.00	0.00
2000-2002	1595	77.62	2.07	17.68	1.44	0.88	0.31
2003-2005	1872	79.59	0.59	14.42	2.08	3.31	0.00
Total	12676	70.42	1.03	16.38	3.08	9.03	0.06
Kidney cancer							
1973-1975	162	38.27	0.00	20.99	8.64	32.10	0.00
1976-1978	162	51.23	0.00	17.90	4.32	26.54	0.00
1979-1981	149	51.68	0.00	18.12	5.37	24.83	0.00
1982-1984	182	55.49	0.00	19.23	2.75	22.53	0.00
1985-1987	286	62.24	0.00	18.18	2.45	16.78	0.35
1988-1990	434	57.60	0.23	32.03	5.99	4.15	0.00
1991-1993	542	60.33	0.92	35.42	3.32	0.00	0.00
1994-1996	560	58.75	0.71	37.32	3.21	0.00	0.00
1997-1999	781	63.12	0.51	34.70	1.66	0.00	0.00
2000-2002	1098	69.40	1.18	27.50	1.09	0.73	0.09
2003-2005	1455	72.92	0.48	22.20	1.44	2.96	0.00
Total	5811	64.09	0.59	27.76	2.56	4.99	0.02

^a^ The figure included the death certificate notification (DCN) and the death certificate only (DCO) cases before 1987; It was only the DCO cases after 1987.

### Statistical analysis

In this study, 3-year age-adjusted incidence rates of urinary bladder and kidney cancers and calendar period from 1973-1975 to 2003-2005 were calculated by direct standardization with the World Standard Population adjusted by Doll R in 1966 [10]. The annual percentage change (APC) for incidence rates was used to examine the secular trends [11]. The natural logarithm of the rates were fitted by a regression line (y= α+βx+ɛ, where y = ln (rate) and x = calendar year). We used the weighted least squares method to estimate the parameter β and the weight is the inverse of the variance of ln (rate) which is approximately equal to the number of cases [11]. The APC was calculated as 100×(e^β^- 1) *w*ith the 95% confidence interval calculated by the methods for population-based cancer statistics recommended by the National Cancer Institute (USA) [12].

An age-period-cohort model was further used to analyze bladder (ICD 10: C67) and kidney (ICD 10: C64) cancer incidences and provided a simultaneous assessment of the contribution of age, period and cohort effects on observed time trends. Ages included in the model were truncated and categorized into eleven 5-year age groups (30–34, 35–39 ... 80–84) since bladder and kidney cancers had few cases under the age of 30. Years of diagnosis were also truncated and divided into six 5-year calendar periods (1976–1980, 1981–1985 ... 2001–2005). As a result, sixteen 10-year overlapping birth cohorts were obtained by subtracting the 5-year age bands from the 5-year periods of diagnosis. In order to solve the problem of overlap, midpoints of each birth cohort bands were extracted to yield sixteen new groups (1896 , 1901 , 1906 , 1911 , 1916 , 1921 , 1926 , 1931 , 1936 , 1941 , 1946, 1951 , 1956 , 1961 , 1966 , 1971 ) .

We used a novel method to fit the age-period-cohort model—namely the intrinsic estimator (IE) method [13-15], which is aimed to deal with the non-identifiability problem [16,17]. The regression coefficients and its standard errors were computed using the STATA version 11 (STATA Corporation, College Station, TX, USA). For all the analysis in this study, two-sided *P*-values <0.05 were considered statistically significant.

## Results

Between 1973 and 2005, there were 12,676 cases for urinary bladder cancer (ICD 10: C67), 5,811 cases for kidney cancer (ICD 10: C64), 631 cases for renal pelvis cancer (ICD 10: C65) and 493 cases for ureter cancer (ICD 10: C66) identified by the Shanghai Cancer Registry. During this period, the diagnosis of 70.42% patients was based on pathology and the proportion of death certificate cases was 9.03% for urinary bladder cancer. For kidney cancer, 64.09% of patients was diagnosed based on pathology and 4.99% as the death certificate cases. During the 33-year period, there was a sharp increase in the percentage of HV for these two cancers, while the death certificate cases experienced a drastic reduction (Table 1). The above messages indicated that our data was considerably reliable in this study. 

### Incidence trends

**Figure 1:**
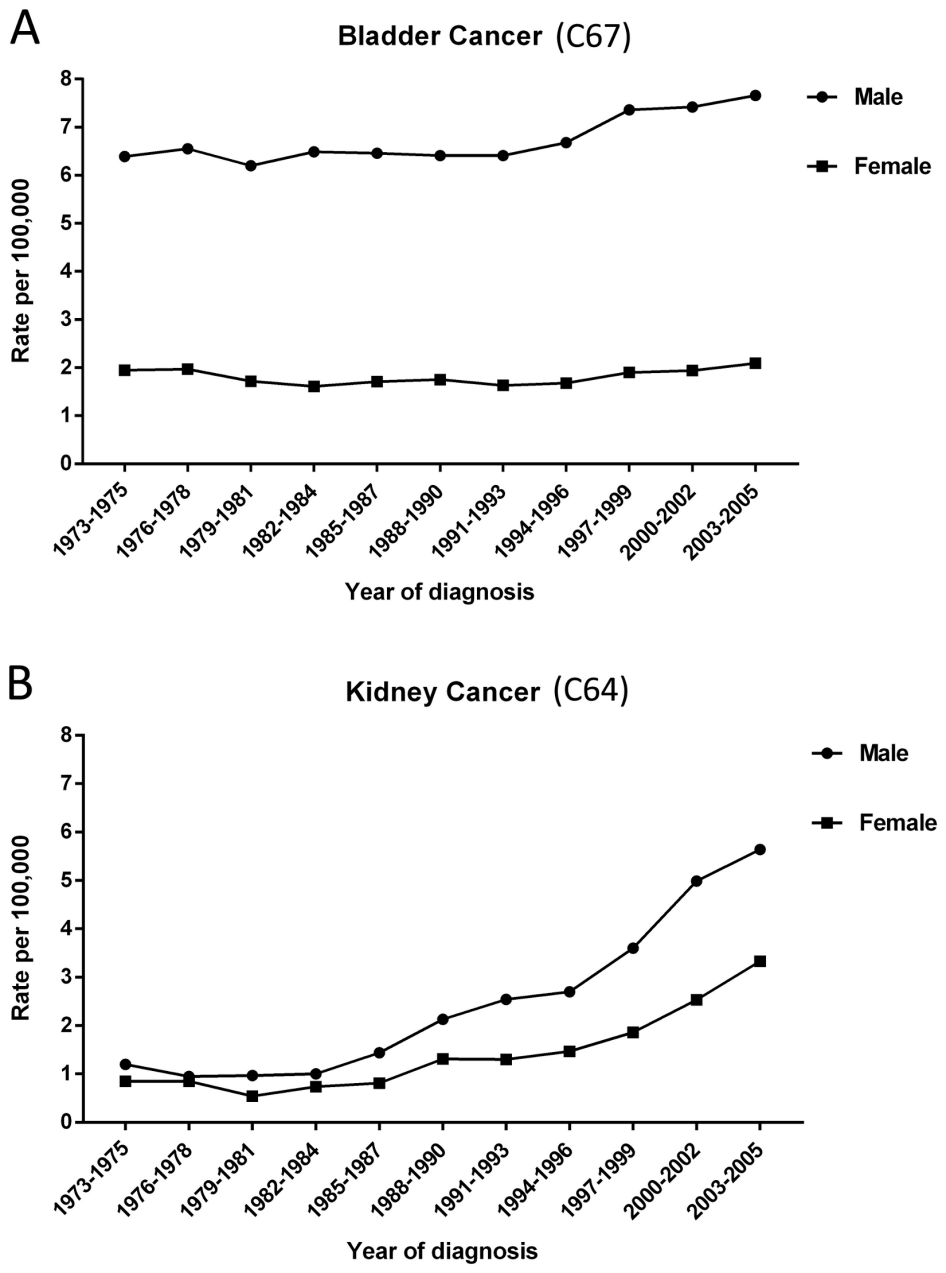
Trends in incidence rates of cancers of bladder and kidney in urban Shanghai, 1973-2005.

The age-standardized incidence rates of bladder cancer were around 7.66 per 100,000 for male and 2.09 per 100,000 for female during 2003 to 2005. During this 33-year period, the overall age-standardized incidence rates had increased annually by 0.62% (P <0.05) and 0.33% (P >0.05) for male and female respectively (Figure 1). The age-standardized incidence rates of kidney cancer during 2003 to 2005 were 5.64 per 100,000 for male and 3.33 per 100,000 for female which exceeded the rate of bladder cancer among females. During the years of 1973 to 2005, kidney cancer had a most dramatically increase with an APC of 6.98% (P <0.0001) for male and 5.93% (P <0.0001) for female (Figure 1). Besides, age-standardized incidence rates for cancers of kidney, renal pelvis and ureter (ICD 10: C64-66) together showed dramatically increase for both genders (Table 2).

**Table 2:** Incidence trends for cancers of bladder, kidney, renal pelvis and ureter by gender in urban Shanghai (1973-2005).

	**1973-1975**		**2003-2005**					
**Sex**	**Cases**	**Rate^*a*^**	**Cases**	**Rate^*a*^**	**Percent Change^*b*^ (%)**	**APC^*c*^**	***P* value^*d*^**	**95% CI**
Bladder (C67)								
Male	470	6.39	1421	7.66	19.87	0.62	0.0017	(0.25, 1.00)
Female	181	1.95	451	2.09	7.18	0.33	0.1958	(-0.18,0.85)
Kidney (C64)								
Male	91	1.20	919	5.64	370.00	6.98	<0.0001	(6.28, 7.69)
Female	71	0.85	536	3.33	291.76	5.93	<0.0001	(5.04, 6.83)
Kidney, renal pelvis and ureter (C64-66) **^*e*^**								
Male	109	1.41	1044	6.35	350.35	6.43	<0.0001	(5.82, 7.04)
Female	77	0.92	640	3.79	311.96	5.94	<0.0001	(5.14, 6.75)

APC, annual percentage change; ICD-10, International Classification of Diseases, Tenth Revision; CI, confidence interval;

^*a*^ Age-standardized rate to the World Standard Population

^*b*^ Percent change between 1973 and 2005 was calculated by the age-standardized rate (world population)

^*c*^ APC was calculated based on age-standardized (world population, per 100,000) incidence rate

^*d*^ *P* value is calculated for the APC^d^

^*e*^ The category *kidney, renal pelvis and ureter* (*C64-66*) is based on the list of the cancer sites from the cancer dictionary available in the CLOBOCAN database (IARC).

### Age-period-cohort analysis

This IE method was used to disentangle the relative contributions of chronological age, historical period, and birth cohort on the incidences of urinary bladder and kidney cancers in urban Shanghai from 1975 to 2005. Age-period-cohort analysis showed apparently increasing trends of age and period effects but generally decreasing cohort effects for bladder and kidney cancers.

For bladder cancer, the age was an important factor contributing to the incidence trend. With it increased from 30 to 84 years old, the relative risk of bladder cancer showed a remarkable rise for both male and female. The risk in the aged 80-84 group in male was about five times higher than that for all ages combined. For female, it was about four times higher compared with all ages combined (Table 3). When we considered period effects for bladder cancer, the curve of relative risk also increased dramatically after the relatively mild rise before 1996. Moreover, the results indicated that for both genders the risks of bladder cancer incidence were more than doubled in 2001–2005 as compared to 1976–1980. It was calculated as the difference in the coefficients for two periods of 2001–2005 and 1976–1980, which is e^0.444 to−0.348^=2.21 in male and e^0.503 to −0.253^ =2.13 in female (Table 3). For the birth cohort effect, the most notable peak occurred in 1906 for both genders and the relative risks were both about two times higher than all birth cohorts combined by gender. Although the incidence risk of birth cohort showed a general decline for both genders, there still existed some differences between male and female. For men who born in 1956 and 1971, it showed two slight rising waves by further examining the curve of cohort effects. However, for women, this general decline (certain increase occurred in the first three cohorts) ceased until the latest birth cohort of 1971 (Table 3). 

**Table 3:** Estimated regression coefficients and standard errors for bladder cancer (C67) incidence rate by gender.

**Intercept**	**s.e.**	**Age groups**	**Effect**	**s.e.**	**Periods**	**Effect**	**s.e.**	**Cohorts**	**Effect**	**s.e.**
Male										
-9.077	0.035	30-34	-2.379	0.150	1976-1980	-0.348	0.041	1896	0.700	0.161
		35-39	-1.705	0.104	1981-1985	-0.252	0.035	1901	0.711	0.093
		40-44	-1.107	0.085	1986-1990	-0.135	0.030	1906	0.689	0.069
		45-49	-0.708	0.072	1991-1995	0.027	0.029	1911	0.622	0.057
		50-54	-0.173	0.062	1996-2000	0.263	0.030	1916	0.493	0.051
		55-59	0.188	0.054	2001-2005	0.444	0.032	1921	0.408	0.046
		60-64	0.611	0.044				1926	0.252	0.049
		65-69	0.979	0.037				1931	0.043	0.055
		70-74	1.255	0.035				1936	-0.194	0.065
		75-79	1.438	0.039				1941	-0.396	0.082
		80-84	1.601	0.048				1946	-0.492	0.089
								1951	-0.429	0.088
								1956	-0.339	0.091
								1961	-0.841	0.133
								1966	-0.742	0.204
								1971	-0.485	0.396
Female										
-10.359	0.052	30-34	-2.111	0.224	1976-1980	-0.253	0.061	1896	0.340	0.233
		35-39	-1.456	0.156	1981-1985	-0.267	0.053	1901	0.557	0.135
		40-44	-1.290	0.147	1986-1990	-0.158	0.046	1906	0.778	0.097
		45-49	-0.514	0.110	1991-1995	-0.055	0.046	1911	0.674	0.083
		50-54	-0.138	0.096	1996-2000	0.229	0.046	1916	0.666	0.073
		55-59	0.164	0.083	2001-2005	0.503	0.049	1921	0.416	0.069
		60-64	0.530	0.068				1926	0.221	0.074
		65-69	0.944	0.056				1931	0.051	0.082
		70-74	1.166	0.053				1936	-0.072	0.097
		75-79	1.366	0.057				1941	-0.346	0.127
		80-84	1.339	0.070				1946	-0.329	0.135
								1951	-0.379	0.137
								1956	-0.581	0.151
								1961	-0.601	0.195
								1966	-0.852	0.309
								1971	-0.541	0.581

For kidney cancer, the risk continued to rise rapidly until 70-74 years old group for both male and female, with a slight decline appearing afterwards. For age effects, the highest risk of kidney cancer incidence were more than doubled for male and almost 90% higher for female in comparison with all ages combined (calculated as e^0.840^ =2.32 in male and e^0.629^ =1.88 in female) (Table 4). For period effect of kidney cancer, a dramatically increase occurred after the group of 1981-1985 for both genders, indicating that there may be common risk pattern for both genders after the group of 1981-1985. The increased risk from 1976-1980 to 2001-2005 was more than nine fold for male and nearly sevenfold for female (calculated as the difference in the coefficients for two periods of 2001–2005 and 1976–1980, which is e ^1.204 to -1^.^049^ = 9.52 in male, and e ^1.100 to -0.798^ = 6.67 in female) (Table 4). Different from the age and period effects, the cohort effect for kidney cancer showed more complex changes between generations to generations. If we simply took 1936 birth cohort as the cut-off point, we would find that the incidence risk for the subsequent generations after 1936 was much lower than those who were born before 1936. Furthermore, for male, there was a marked peak occurring in the generation born in 1906 and the relative risk nearly doubled the risk of all birth cohorts combined. The birth cohort effect for female showed a general decline before 1961 but an apparent rise afterward which was distinct from male. Besides, we could also find dramatic fluctuations in the first several cohorts and a small peak around 1946 for female (Table 4). Additionally, more details about age, period and cohort effects for bladder and kidney cancers could be obtained in Figure S1.

**Table 4:** Estimated regression coefficients and standard errors for kidney cancer (C64) incidence rate by gender.

**Intercept**	**s.e.**	**Age groups**	**Effect**	**s.e.**	**Periods**	**Effect**	**s.e.**	**Cohorts**	**Effect**	**s.e.**
Male										
-10.073	0.068	30-34	-2.118	0.211	1976-1980	-1.049	0.095	1896	0.274	0.691
		35-39	-1.475	0.142	1981-1985	-0.996	0.088	1901	0.563	0.336
		40-44	-0.696	0.108	1986-1990	-0.148	0.060	1906	0.643	0.218
		45-49	-0.146	0.090	1991-1995	0.270	0.052	1911	0.425	0.177
		50-54	0.269	0.081	1996-2000	0.720	0.052	1916	0.328	0.147
		55-59	0.510	0.077	2001-2005	1.204	0.057	1921	0.245	0.122
		60-64	0.595	0.073				1926	0.173	0.109
		65-69	0.786	0.071				1931	-0.096	0.101
		70-74	0.840	0.078				1936	-0.291	0.099
		75-79	0.731	0.094				1941	-0.335	0.103
		80-84	0.704	0.117				1946	-0.347	0.100
								1951	-0.159	0.093
								1956	-0.358	0.100
								1961	-0.295	0.123
								1966	-0.365	0.197
								1971	-0.404	0.407
Female										
-10.592	0.063	30-34	-1.316	0.187	1976-1980	-0.798	0.098	1896	-0.079	0.628
		35-39	-1.112	0.149	1981-1985	-0.759	0.092	1901	0.452	0.306
		40-44	-0.746	0.129	1986-1990	-0.233	0.070	1906	0.383	0.225
		45-49	-0.143	0.104	1991-1995	0.099	0.062	1911	0.103	0.193
		50-54	0.163	0.095	1996-2000	0.591	0.057	1916	0.390	0.144
		55-59	0.313	0.089	2001-2005	1.100	0.059	1921	0.287	0.119
		60-64	0.516	0.081				1926	0.255	0.108
		65-69	0.456	0.081				1931	0.149	0.101
		70-74	0.629	0.083				1936	-0.161	0.107
		75-79	0.649	0.096				1941	-0.198	0.115
		80-84	0.590	0.119				1946	-0.129	0.112
								1951	-0.314	0.112
								1956	-0.462	0.116
								1961	-0.444	0.142
								1966	-0.206	0.187
								1971	-0.025	0.310

## Discussion

The present study included 12,676 and 5,811 incidence cases of bladder and kidney cancer recorded in the population-based Shanghai Cancer Registry from 1973 through 2005. There was a relatively mild increase in age-standardized incidence rate of bladder cancer, but a dramatic rise for kidney cancer. From the age-period-cohort analysis, results indicated increasing curves of age and period effects but generally decreasing cohort effects both for bladder and kidney cancers. 

For urinary bladder cancer, most Western countries showed much higher incidence rates compared with us. In 2008, the age-standardized incidence rates (age-adjusted to the world-standard population) in USA for all races were 21.1 per 100,000 in men and 5.8 per 100,000 in women which were nearly fourfold than that of both male and female in China [1]. Similarly in Europe, the age-standardized incidence rates (age-adjusted to the world-standard population) were threefold and twofold than that of male and female respectively in China [1]. In USA, the incidence trend in male showed a significant increase during 1975 to 1987 (APC=1.00%) and became stable afterwards. For female, this mild rise (APC=0.20%) was continuing until 2003 [18]. This mild increase in female in USA was quite similar with the trend of female in Shanghai during 1973 to 2005 (APC=0.38%). 

The incidence rate of kidney cancer showed a regional disparity and race difference. Between 1973-1997 and 1988-1992, kidney cancer experienced global increase among men and women in all regions and ethnic groups, with a few exceptions, mostly in Scandinavian countries [19] In 1988-1992, kidney cancer incidence rates (age-adjusted to the world-standard population) were highest in Bas-Rhin (France) (14.5 per 100,000 in men and 6.9 per 100,000 in women) and lowest in Bombay, India (1.8 and 0.8, respectively) [19]. In the United States, the Asian and Pacific Islander showed the lowest rates (4.7 per 100,000 in men and 2.2 per 100,000 in women) than other races such as White, Black, American Indian/Alaska Native and Hispanic during 1998-2002 [20]. The incidence rates for kidney cancer rose consistently over time, with the increases more rapid among blacks than whites and a shift from a predominance among whites to blacks [20]. Although the incidence rate of kidney cancer in urban Shanghai was lower than that in some Western countries historically, the rates increased more sharply in Shanghai during the past decades [19]. 

By the age-period-cohort analysis, we found that the age effects for bladder and kidney cancers showed different increasing curves. Incidence risk of bladder cancer showed a moderate but linear increase up to age of 55 years old, with a greater increase thereafter. However, the age effect of kidney cancer rose sharply before 60 years old but showed a generally mild change afterwards. Moreover, the highest incidence risk occurred around 70-74 years old group for kidney cancer but 80-84 years old group for bladder cancer. Bladder and kidney cancers increased at different rates with age. It is possible that certain molecular and physiological changes specific to each cancer type might be affecting these disparate rates of change. Of note, kidney cancer may occur and dramatically rise at a relatively early age in comparison with bladder cancer. Thus, for future surveillance and screening on kidney cancer, more attentions should be paid to individuals after 35 years old. By controlling the age and birth cohort effects, the contribution of period effect can be better elucidated. Breakthroughs in medical technology and diagnostic techniques might be the main factor which influences period effect of certain cancer [21]. Since 1980s, with more advanced medical technology and facilities introduced to Shanghai, increased number of cancer patients had been detected. The wide use of cystoscopy might lead to increased examinations for urinary bladder cancer which enhanced the chance of early diagnosis. It had also shown that early detection of kidney and bladder cancers had risen with gradually increased use of imaging procedures during the study periods, such as ultrasonography, computed tomography, and magnetic resonance imaging [22,23]. These advanced diagnostic techniques, to some extent, contributed to the increased period effects on bladder and kidney cancers. Furthermore, although bladder and kidney cancers both showed increasing trends during 1976 to 2005, the relative risk of kidney cancer increased more sharply than that of bladder cancer. 

Additionally, period effect subsumes a complex set of historical events including public health efforts and certain environmental risk factors which affect incidence of all society members [24]. Cigarette smoking, as well as passive smoking, which has possibilities to influence those of all ages, has consistently been observed to be a common risk factor for bladder and kidney cancers [25]. In 1984, China had approximately 250 million smokers, with a prevalence of 61% in men and 7% in women [26]. Since then, smoking prevalence in China became much higher among men, up to 63% in 1996 and 66% in 2002. Of the nonsmoker (mostly women and children) in 1996, 53.5% reported passive smoke exposure [27]. The number of smokers had approximately increased to 350 million in 2002 [28]. An estimated 72% of Chinese individuals over the age of 15 years had been exposed to tobacco, including those exposed to second-hand smoke [27]. Lack of powerful tobacco control activities as well as other effective and comprehensive public health efforts might partly explain the continuously rising period effects of bladder and kidney cancers in Shanghai.

Cohort effect reflects variations in incidence across groups of memberships born in the same year or years. It is well known that the significance of early life exposures in explaining the susceptibility to certain cancer and incidence later in the adulthood. The sharply declining trends of cohort effects for bladder and kidney cancers both ceased and began generally leveling off around 1940s. The most notable peak occurred in the birth cohort of 1906 for bladder and kidney cancers. In the early period of the twentieth century in China, the poorly living conditions and chronic malnutrition might be early life exposures to later development of bladder and kidney cancers for the specific birth cohort. Moreover, some unknown exposures earlier in life, such as exposure to cigarette smoking, childhood obesity and physical inactivity, may partly explain other slight changes of birth cohort effects for these two cancers in Shanghai [29-32]. In addition, the observed cohort effect may have bias due to miss-caught cases (before 1973) in oldest birth cohort.

This study also possessed some strengths and limitations. Firstly, we acknowledged that several limitations might exist in our study. One unavoidable limitation in this study is the potential information bias by under-diagnosis/reporting (lack of diagnostic capabilities) in the early years, which might somewhat contribute to the described rise in incidence, especially for kidney cancer, during the past 33 years. It is a question and a limitation for any cancer registry if they did a time trend analysis of incidence because of development of diagnosis and medical techniques. For example, in the last 2 decades, the use of abdominal imaging has also increased in the United States, leading to more renal cancers being detected at local or regional stages of disease [33]. Since the incidence of cancer presenting at a distant stage has not declined in the United States [33], this is thought to be a real increase, not only due to changes in the way the disease is diagnosed [34]. However, we failed to find any publications or resources for identifying the exact contributions of the information bias on the cancer trends. Moreover, increasing exposures to potential risk factors could also affect incidence trend of these two cancers in Shanghai, such as tobacco consumption, obesity and hypertension, etc. [35,36] which may contribute to the observed rise in our study. It is also possible that the trend toward a more westernized diet in Shanghai plays a role, since high consumption of meat and fat has been linked to kidney cancer risk [19]. In addition, the incidence trends of these two cancers in urban Shanghai from 1973 to 2005 were also in consistent with numerous studies on these cancers in Western populations. Therefore, we do think that this kind of rise in incidence may be caused by the combined effects of aging of population, improved medical techniques and the increasing exposures to certain risk factors. Furthermore, the natural characteristic of age-period-cohort analysis is still descriptive, not analytic research. As lacking further knowledge about the comprehensive effects of the risk factors of bladder and kidney cancers in urban Shanghai, explicit explanations of some results from age-period-cohort analysis were still challenging. This descriptive study could only try to offer some clues about risk factors of these cancers which were reflected by the effects of age, period and birth cohort illustrated in our study. However, it is the first time to do incidence trend analysis of urinary bladder and kidney cancers during past 33-years using the population-based registration data in China, as well as in other Asian countries. It could not only provide the upcoming studies with some basic data about urinary bladder and kidney cancers in Shanghai, but guide our future work for deeply explorations of the risk factors for these two cancers. Our present study also supplies an opportunity to make a comparison between Eastern and Western countries on bladder and kidney cancer incidences. And most importantly, it could guide the local health officials to make current efforts on prevention and surveillance of bladder and kidney cancers. Of note, we should pay more attention to the fast rise of kidney cancer incidence, especially for the middle-aged and high risk individuals with some potential risks. Besides, the novel IE method which enabled us to explore the time trends of the incidence rates without relying on any priors could provide more accurate estimators than some traditional methods [13]. 

In summary, our study showed mildly increasing incidence trends in urinary bladder cancer but sharply increased incidence rate in kidney cancer in Shanghai, China. Aging of population, advancing diagnostic techniques and exposing to some unknown risks may together contribute to the increasing incidence trends of bladder and kidney cancers. Further studies should aim at exploring those potential risk or protective factors and reducing the incidence rates of these cancers. 

## Supporting Information

Figure S1 **Age, period and cohort effects for bladder (C67) and kidney (C64) cancers by gender.** Note: The reference group for the cohort coefficients is the mean influence of all cohorts combined, and the reference groups for the period and age coefficients are the mean influence of all periods and ages combined, respectively. For example, the value of 0.711 for the 1901 birth cohort of male indicates that membership in this cohort nearly doubled the risk (e^0.711^= 2.04) of incidence of bladder cancer compared to all cohorts combined, which is independent of period and age effects.
(TIF)
