# Supplementary figures and images for: Incidence Trends of Urinary Bladder and Kidney Cancers in Urban Shanghai, 1973-2005
Source: PLoS One. 2013 Dec 4;8(12):e82430. doi: 10.1371/journal.pone.0082430 (PMC3853415; doi:10.1371/journal.pone.0082430)

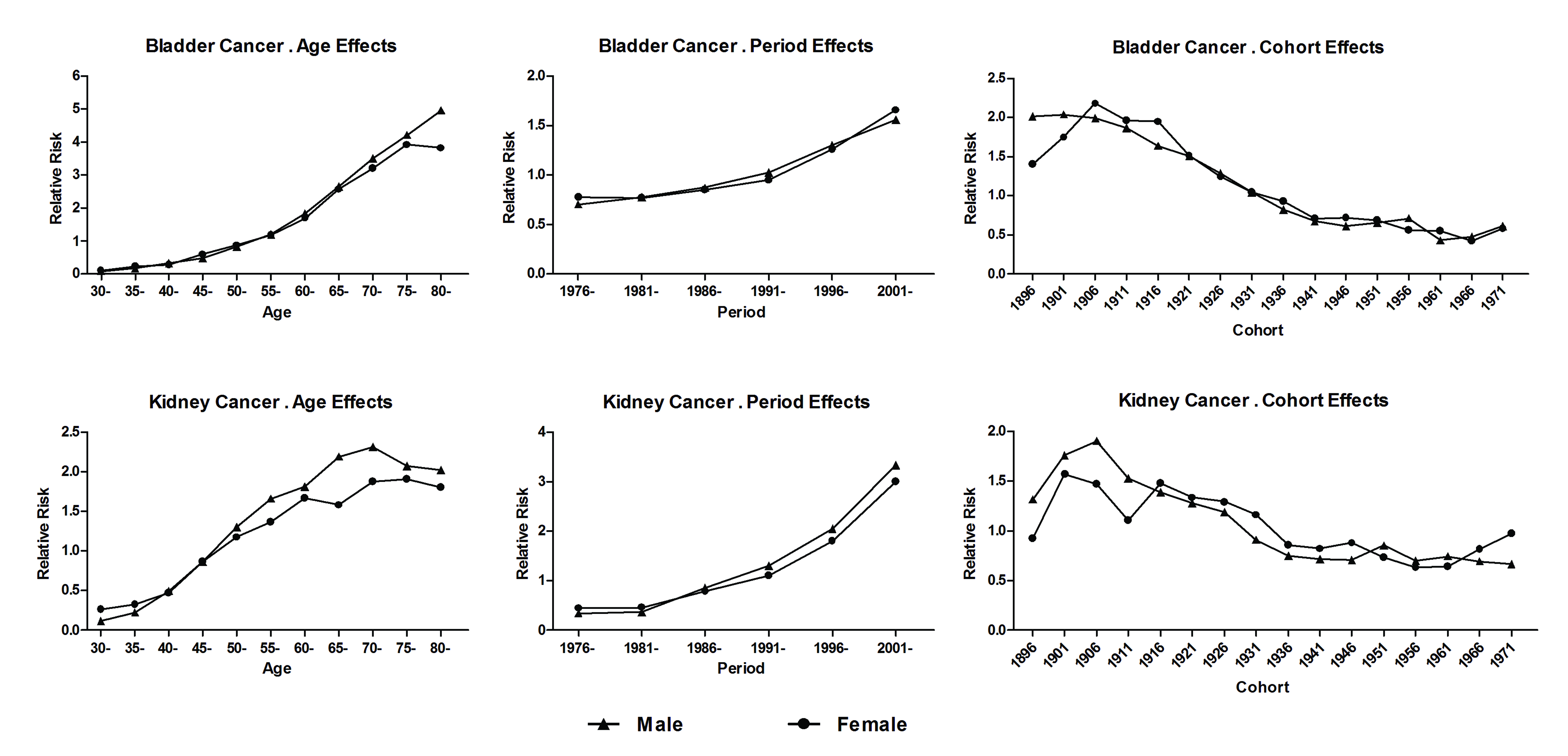

Supplement: Figure S1 — Age, period and cohort effects for bladder (C67) and kidney (C64) cancers by gender. Note: The reference group for the cohort coefficients is the mean influence of all cohorts combined, and the reference groups for the period and age coefficients are the mean influence of all periods and ages combined, respectively. For example, the value of 0.711 for the 1901 birth cohort of male indicates that membership in this cohort nearly doubled the risk (e0.711= 2.04) of incidence of bladder cancer compared to all cohorts combined, which is independent of period and age effects. (TIF) [file pone.0082430.s001.tif]
